# Supplementary material for: The breast cancer oncogene IKKε coordinates mitochondrial function and serine metabolism
Source: EMBO Rep. 2020 Aug 11;21(9):e48260. doi: 10.15252/embr.201948260 (PMC7116048; doi:10.15252/embr.201948260)
Supplement: Supplementary file 1 — Expanded View Figures PDF [file EMBR-21-e48260-s001.pdf]

Expanded View Figures

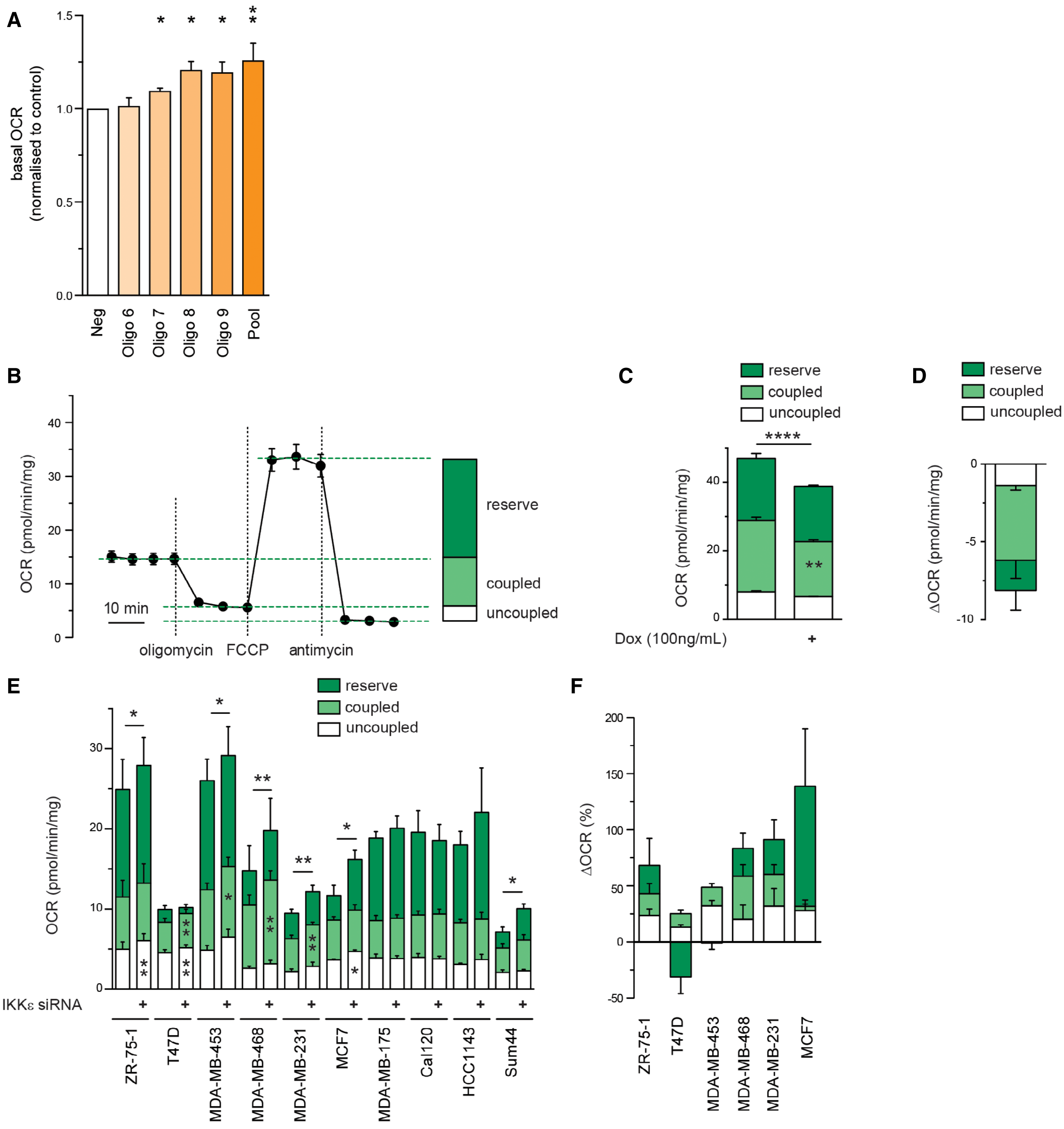

Figure EV1.

Figure EV1. The effect of IKKε on mitochondrial respiration.

A Effect of individual *IKBKE* (IKKε) siRNA oligos on basal OCR in *IKBKE* (IKKε)-silenced MDA-MB-468 cells. Data are normalised to non-silenced control cells (Neg).
B Schematic breakdown of Seahorse analysis, generating OCR curves showing different mitochondrial respiratory functions. Seahorse XF96 analysis determines basal, coupled, uncoupled and reserve respiratory capacities, allowing detailed characterisation of mitochondrial respiratory profiles in various treatment conditions (see Materials and Methods).
C Reserve, coupled and uncoupled respiratory capacity profile of Flp-In 293 HA-IKKε cells treated with doxycycline (Dox) for 16 h.
D Relative individual differences in reserve, coupled and uncoupled respiration in Flp-In 293 HA-IKKε cells treated with doxycycline as in (C). Data presented as changes in OCR (ΔOCR).
E Reserve, coupled and uncoupled respiratory capacity profiles of a panel of *IKBKE* (IKKε)-silenced breast cancer cell lines.
F Relative individual differences in reserve, coupled and uncoupled respiration in indicated *IKBKE* (IKKε)-silenced breast cancer cells, where IKKε had a statistically significant effect on basal OCR (see Fig 3C). Data presented percentage as changes in OCR (ΔOCR).

Data Information: Data were collected using Seahorse XF96e or XF24 analysis (A, E, F) or with Oroboros (C, D), $n \geq 3$, biological replicates $^{*}P < 0.05$, $^{**}P < 0.01$, $^{****}P < 0.0001$. In (A), one-way ANOVA with Fisher's LSD test, and in (C, E), paired *t*-tests or Mann–Whitney test in each cell line between each group (asterisks in individual parameters) and the overall data (asterisks on the top) were used.
Source data are available online for this figure.

Figure EV2. IKKε does not upregulate SBP enzymes through the induction of c-Myc and does not regulate expression of serine catabolising enzyme SHMT2.

A Representative Western blot showing the levels of IKKε, c-Myc and PSAT1 in Flp-In 293 HA-IKKε cells treated with doxycycline for 16 h ($n = 2$ biological replicates).
B Representative Western blot showing the levels of IKKε and SHMT2 in Flp-In 293 HA-GFP or Flp-In 293 HA-IKKε cells treated with doxycycline for 16 h ($n = 2$ biological replicates).
C Representative Western blot showing the levels of IKKε and SHMT2 in a panel of *IKBKE* (IKKε)-silenced breast cancer cell lines.
D Levels of SHMT2 in indicated IKBKE (IKKε)-silenced breast cancer cell lines normalised to Vinculin. Densitometry analysis quantified single sample density as a percentage of total blot density per cell line prior to vinculin normalisation ($n = 3$ biological replicates, mean ± SEM, $^{*}P < 0.05$, two-tailed paired *t*-test was used for all cell lines except MCF7 and Cal120, where Wilcoxon matched-pairs signed rank tests were used).

Source data are available online for this figure.

 © 2020 The Authors

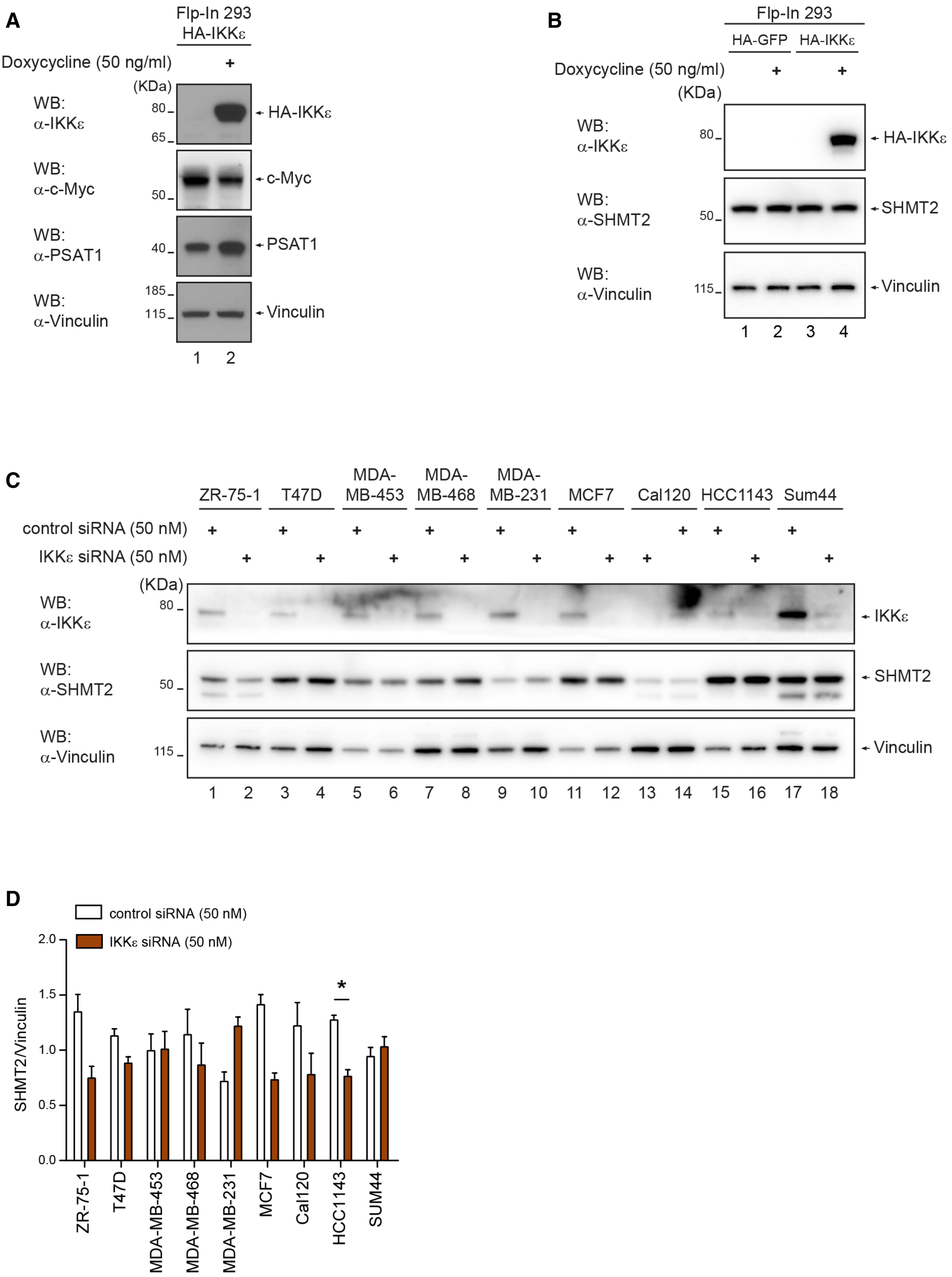
A
Flp-In 293
HA-IKKε
Doxycycline (50 ng/ml) +
(KDa)
WB: α-IKKε
80
65
HA-IKKε
WB: α-c-Myc
50
c-Myc
WB: α-PSAT1
40
PSAT1
WB: α-Vinculin
185
115
Vinculin
1 2
B
Flp-In 293
HA-GFP HA-IKKε
Doxycycline (50 ng/ml) + +
(KDa)
WB: α-IKKε
80
HA-IKKε
WB: α-SHMT2
50
SHMT2
WB: α-Vinculin
115
Vinculin
1 2 3 4
C
ZR-75-1 T47D MDA-MB-453 MDA-MB-468 MDA-MB-231 MCF7 Cal120 HCC1143 Sum44
control siRNA (50 nM)
IKKε siRNA (50 nM)
(KDa)
WB: α-IKKε
80
IKKε
WB: α-SHMT2
50
SHMT2
WB: α-Vinculin
115
Vinculin
1 2 3 4 5 6 7 8 9 10 11 12 13 14 15 16 17 18
D
control siRNA (50 nM)
IKKε siRNA (50 nM)
SHMT2/Vinculin
2.0
1.5
1.0
0.5
0.0
*
ZR-75-1
T47D
MDA-MB-453
MDA-MB-468
MDA-MB-231
MCF7
Cal120
HCC1143
SUM44

Figure EV2.

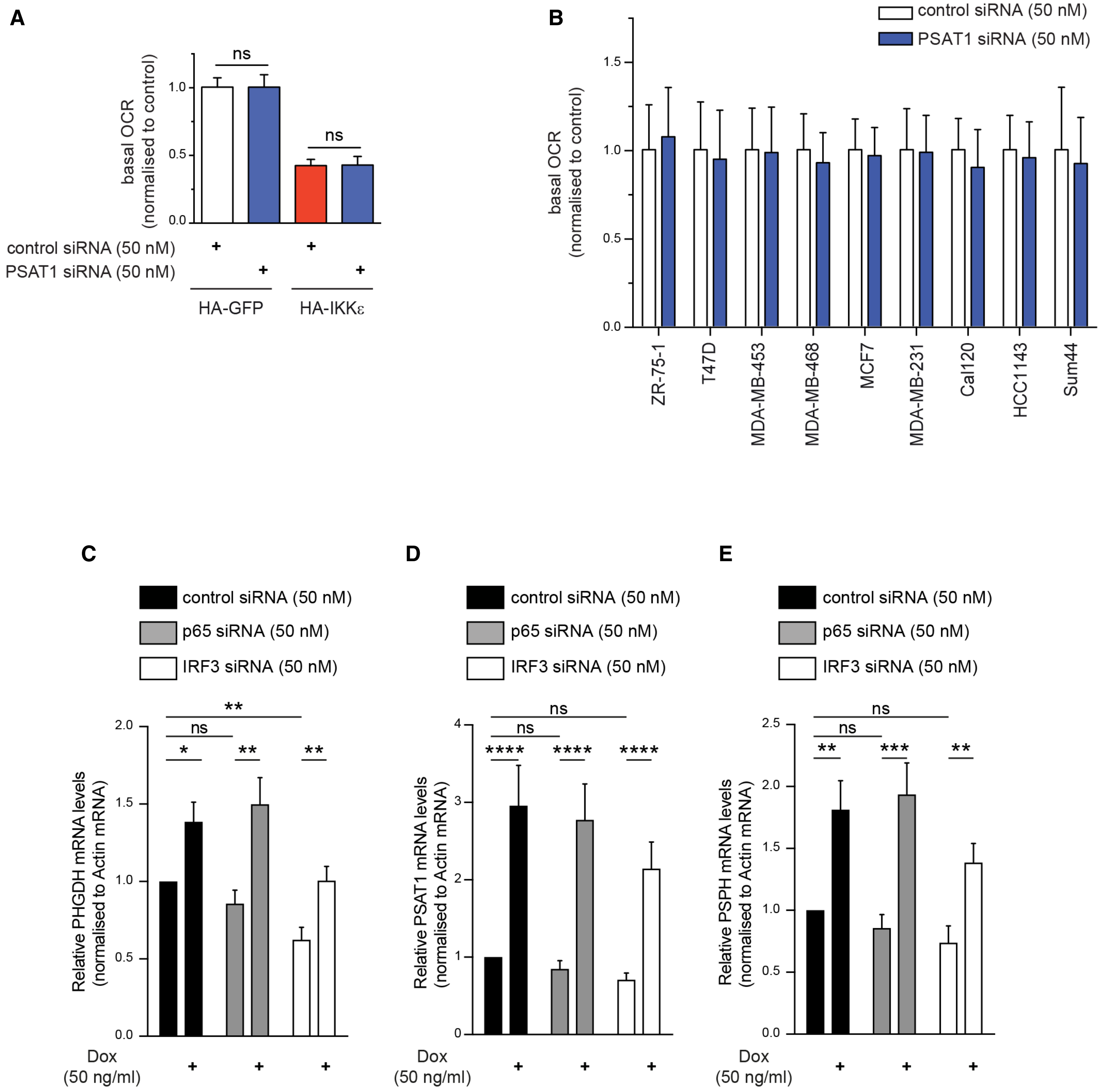

Figure EV3.

 © 2020 The Authors

Figure EV3. siRNA-mediated knockdown of PSAT1 does not affect Oxygen Consumption Rate in Flp-In 293 HA-IKKε cells or IKKε-expressing breast cancer cells and regulation of SBP gene transcription by IKKε is independent of canonical IKKε signalling pathways.

A Basal OCR in *PSAT1*-silenced Flp-In 293 HA-GFP or Flp-In 293 HA-IKKε cells treated with doxycycline (50 ng/ml, 16 h), measured using Oroboros high-resolution respirometry. Data are normalised to non-treated Flp-In 293 HA-GFP control cells. (n = 9 biological replicates).

B Basal OCR in a panel of *PSAT1*-silenced breast cancer cell lines, measured using Seahorse XF96 analysis. Data are first normalised to the total sample protein concentration and then to non-silenced control samples. (n = 3 biological replicates).

C–E qRT–PCR analysis of (C) *PHGDH*, (D) *PSAT1* and (E) *PSPH* mRNA levels in *p65*- or *IRF3*-silenced Flp-In 293 HA-IKKε cells treated with doxycycline (Dox) for 16 h. Data are expressed as fold changes relative to levels in non-silenced, non-treated cells and normalised to *β-Actin*. ($n \geq 4$ biological replicates).

Data Information: In (A–E), data are presented as mean ± SEM. The following statistical tests were applied; in (A) two-way ANOVA with Bonferroni *post hoc* tests, in (B) paired, two-tailed Student's *t*-test. No statistically significant differences were detected. In (C–E), two-way ANOVA with Fisher's LSD tests were performed using log-transformed fold change values,*$P < 0.05$, **$P < 0.01$, ***$P < 0.001$, ****$P < 0.0001$).

Source data are available online for this figure.

Figure EV4. IKKε mediates cytokine secretion but no autocrine effect is responsible for the induction of SBP enzymes.

A–C Representative Western blots showing the levels of IKKε, PHGDH, PSAT1, PSPH, STAT1, phosphorylated STAT1 (Y701), OAS1, p65 and phosphorylated p65 (S486) in (A) Flp-In 293 HA-GFP cells, (B) T47D breast cancer cells and (C) ZR-75-1 breast cancer cells treated for 24 h with media conditioned by Flp-In 293 HA-GFP or Flp-In 293 HA-IKKε cells treated with doxycycline (Dox, 50 ng/ml) for 16 h. Changes in protein expression were compared to changes in Flp-In 293 HA-IKKε cells treated with doxycycline (16 h).

Source data are available online for this figure.

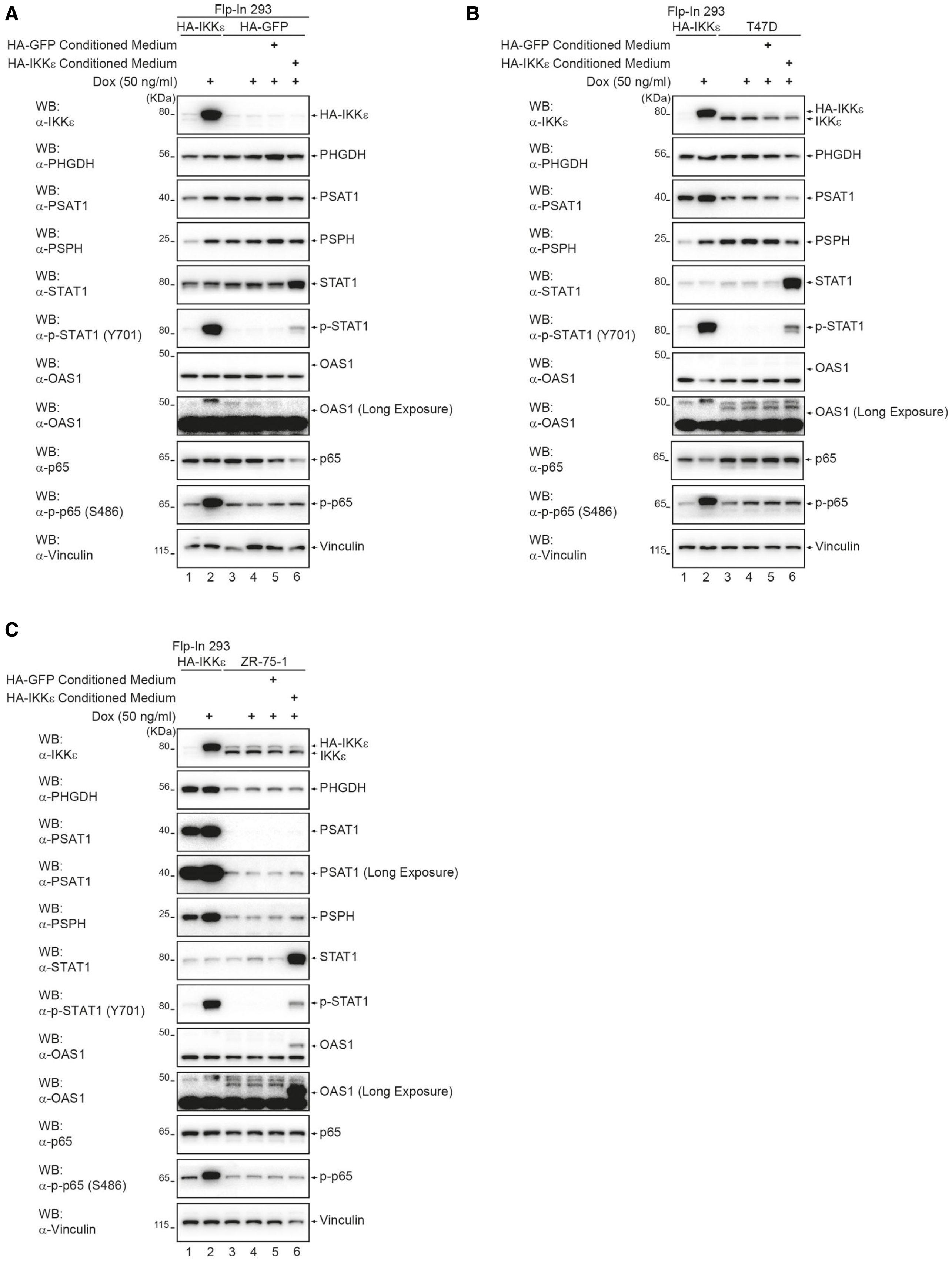

Figure EV4.

 © 2020 The Authors

Figure EV5. The pathophysiological role of IKKε-associated metabolic changes in breast cancer.

A Changes in cell confluency (Δconfluency) over 72 h in a panel of breast cancer cell lines treated with NCT502 or PHGDH inactive control compound (PHGDH IA).
B Correlation of change in extracellular acidification rate (ECAR) (ΔECAR) in a panel of *IKBKE* (IKKε)-silenced breast cancer cell lines, and the Δconfluency upon treatment of the panel of cell lines with NCT502 for 72 h (from Fig EV5A).
C Δconfluency over 96 h in a panel of breast cancer cell lines treated with DON.
D Correlation of ΔECAR in a panel of *IKBKE* (IKKε)-silenced breast cancer cell lines, and the Δconfluency upon treatment of the panel of cell lines with DON for 96 h (from Fig EV5C).
E Δconfluency over 72 h in a panel of breast cancer cell lines treated with CB839.

Data Information: In (A–E), Δconfluency was measured using the IncuCyte Zoom. In (B, D), ΔECAR was measured using Seahorse XF96e or XF24 analysis. Data are $n \geq 3$ biological replicates and are presented as mean ± SEM, *$P < 0.05$, **$P < 0.01$, ***$P < 0.001$, ****$P < 0.0001$. In (A, E), two-way ANOVA with Dunnett's multiple comparison tests was applied, and in (C), multiple *t*-tests were applied. In (B, D), linear regression correlation coefficients (Pearson's *R*, Spearman's rho) are shown.

Source data are available online for this figure.

© 2020 The Authors

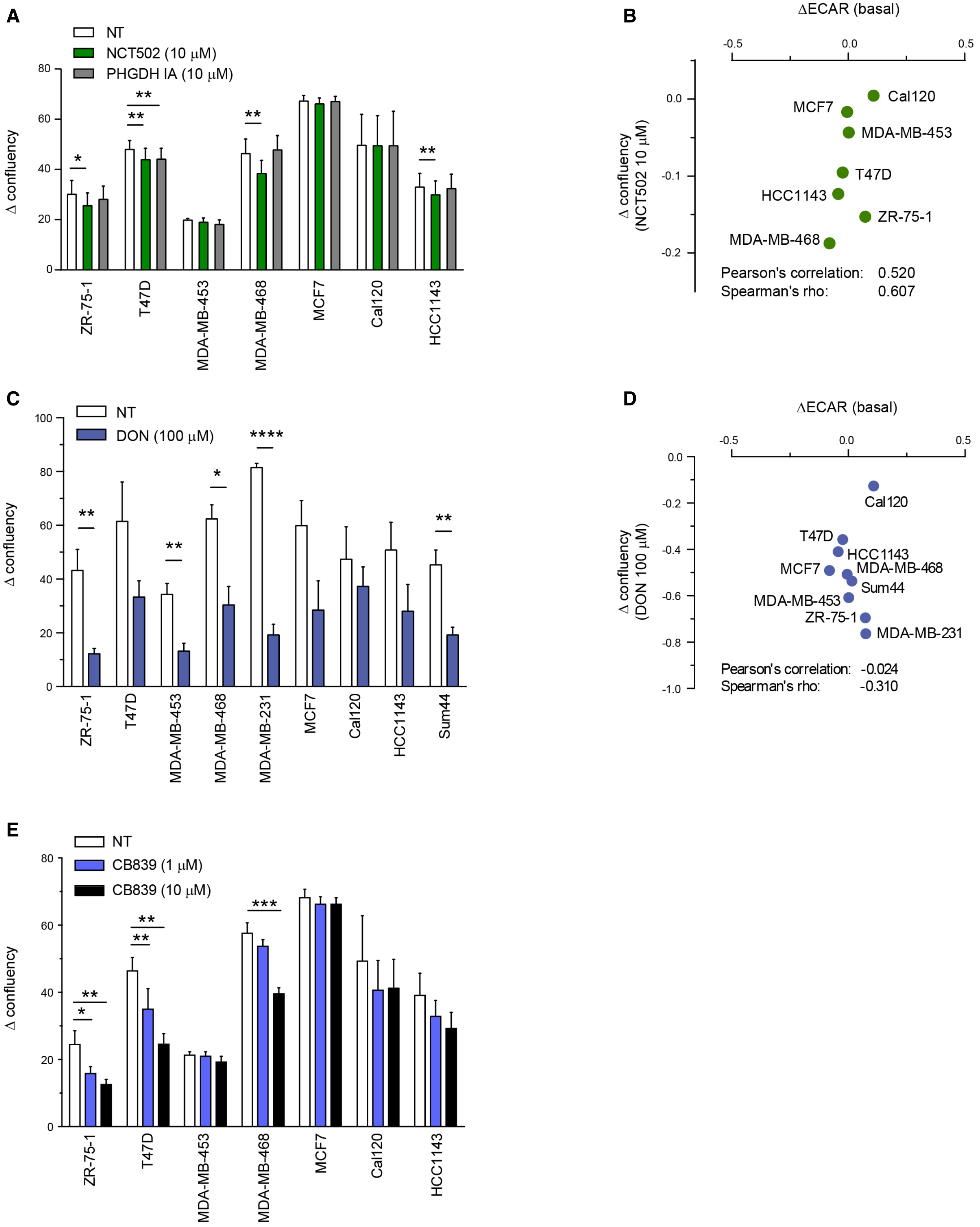

Figure EV5.

© 2020 The Authors
