# Supplementary material for: The breast cancer oncogene IKKε coordinates mitochondrial function and serine metabolism
Source: EMBO Rep. 2020 Aug 11;21(9):e48260. doi: 10.15252/embr.201948260 (PMC7116048; doi:10.15252/embr.201948260)
Supplement: Supplementary file 4 — Source Data for Expanded View [file EMBR-21-e48260-s010.zip › EV_Figure_Source_Data/FigureEV4/Figure EV4 Panel B.pdf]

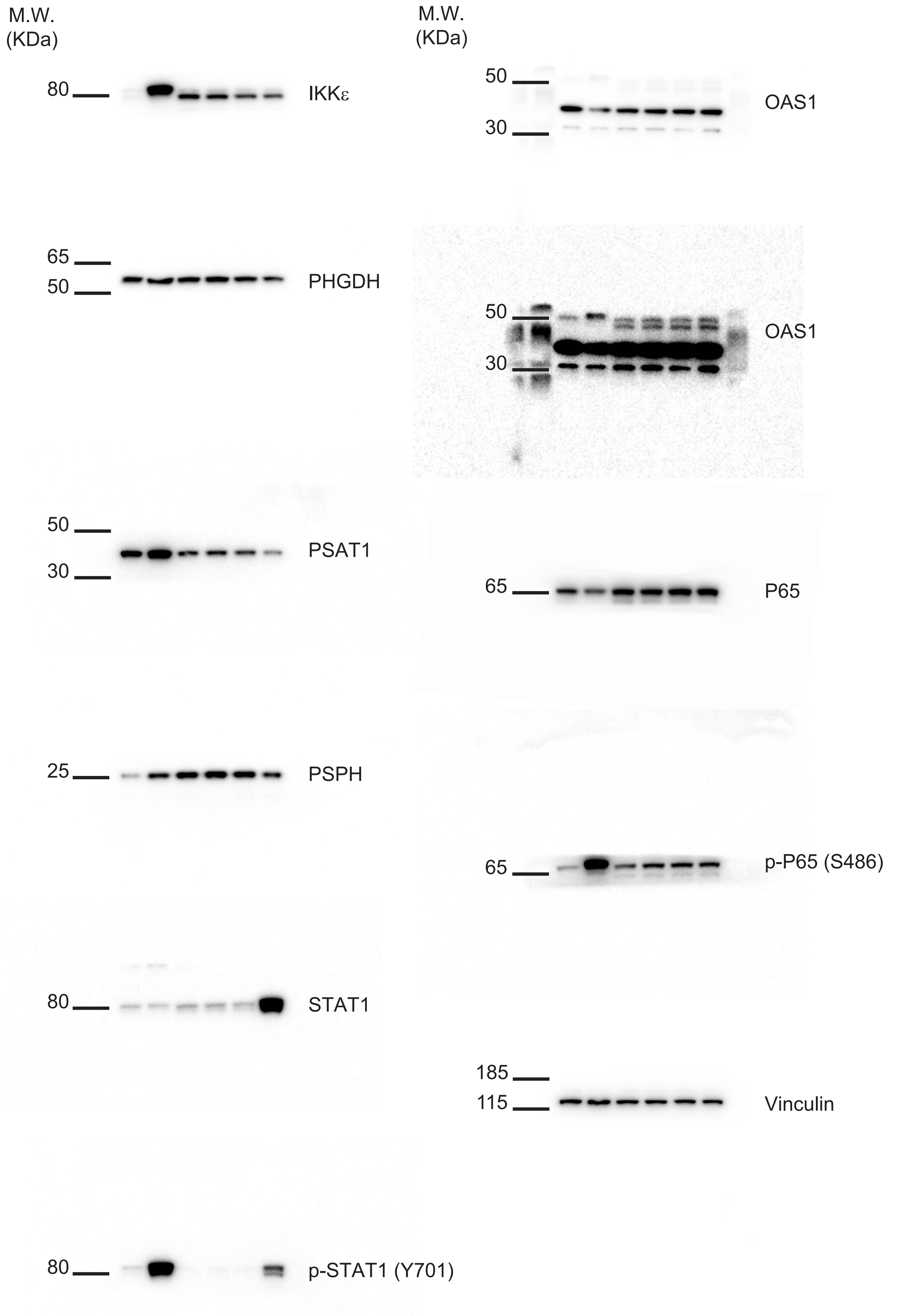
M.W. (KDa)
80
IKKε
65
50
PHGDH
50
30
PSAT1
25
PSPH
80
STAT1
80
p-STAT1 (Y701)
M.W. (KDa)
50
30
OAS1
50
30
OAS1
65
P65
65
p-P65 (S486)
185
115
Vinculin

Figure EV5B
