# Supplementary figures and images for: The breast cancer oncogene IKKε coordinates mitochondrial function and serine metabolism
Source: EMBO Rep. 2020 Aug 11;21(9):e48260. doi: 10.15252/embr.201948260 (PMC7116048; doi:10.15252/embr.201948260)

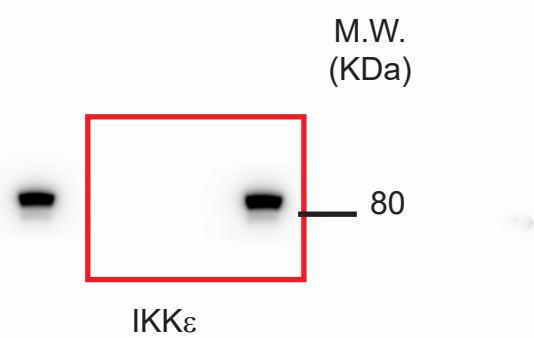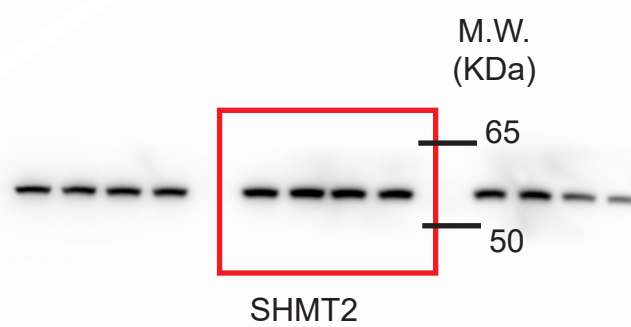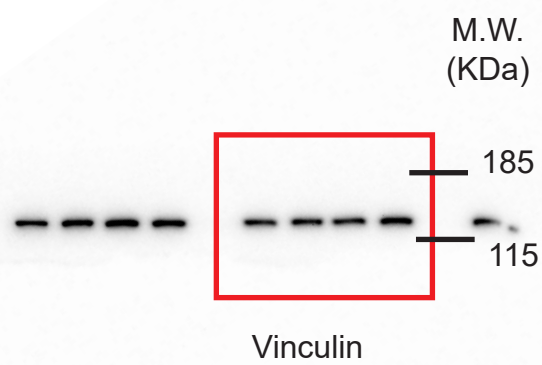

Figure EV2B

Supplement: Supplementary file 4 — Source Data for Expanded View [file EMBR-21-e48260-s010.zip › EV_Figure_Source_Data/FigureEV2/Figure EV2 Panel B.pdf]

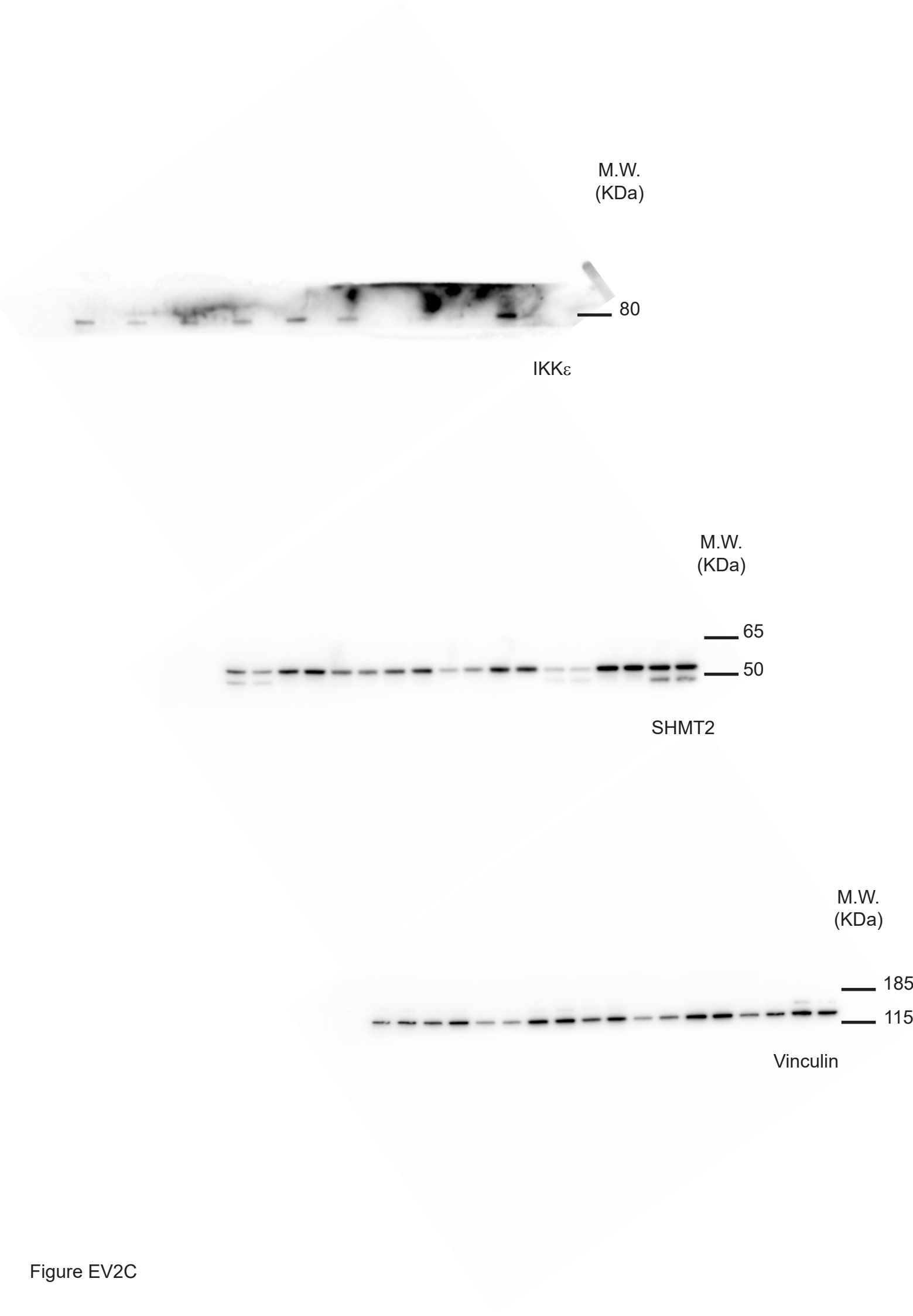

Figure EV2C

Supplement: Supplementary file 4 — Source Data for Expanded View [file EMBR-21-e48260-s010.zip › EV_Figure_Source_Data/FigureEV2/Figure EV2 Panel C.pdf]

Figure EV2A

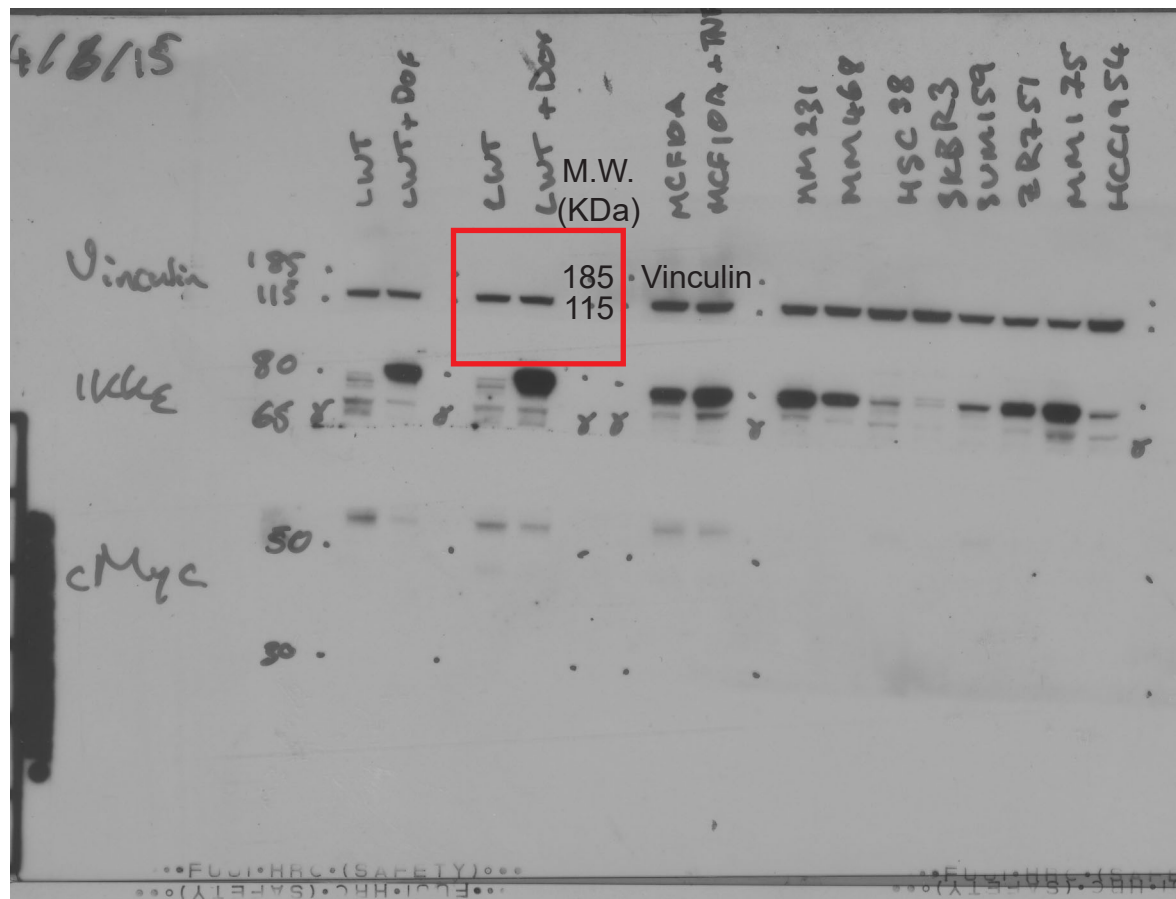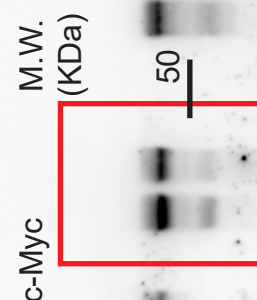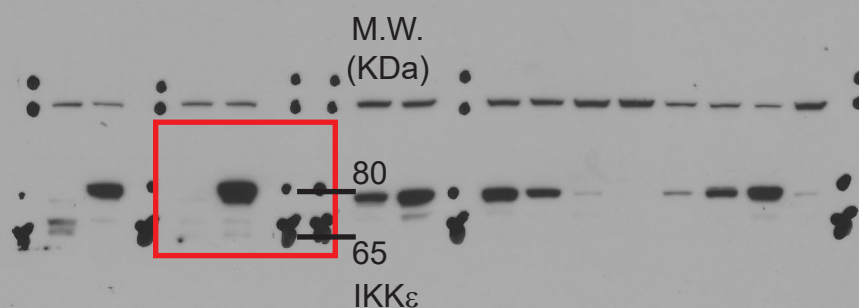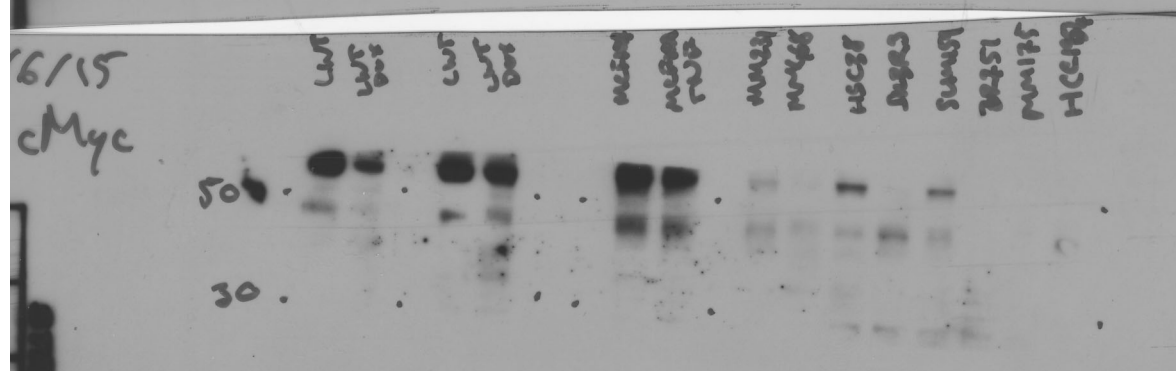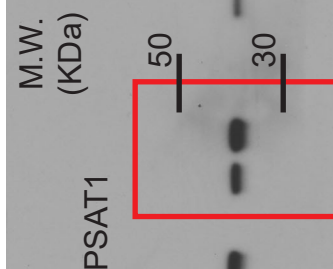

Supplement: Supplementary file 4 — Source Data for Expanded View [file EMBR-21-e48260-s010.zip › EV_Figure_Source_Data/FigureEV2/Figure EV2 Panel A.pdf]

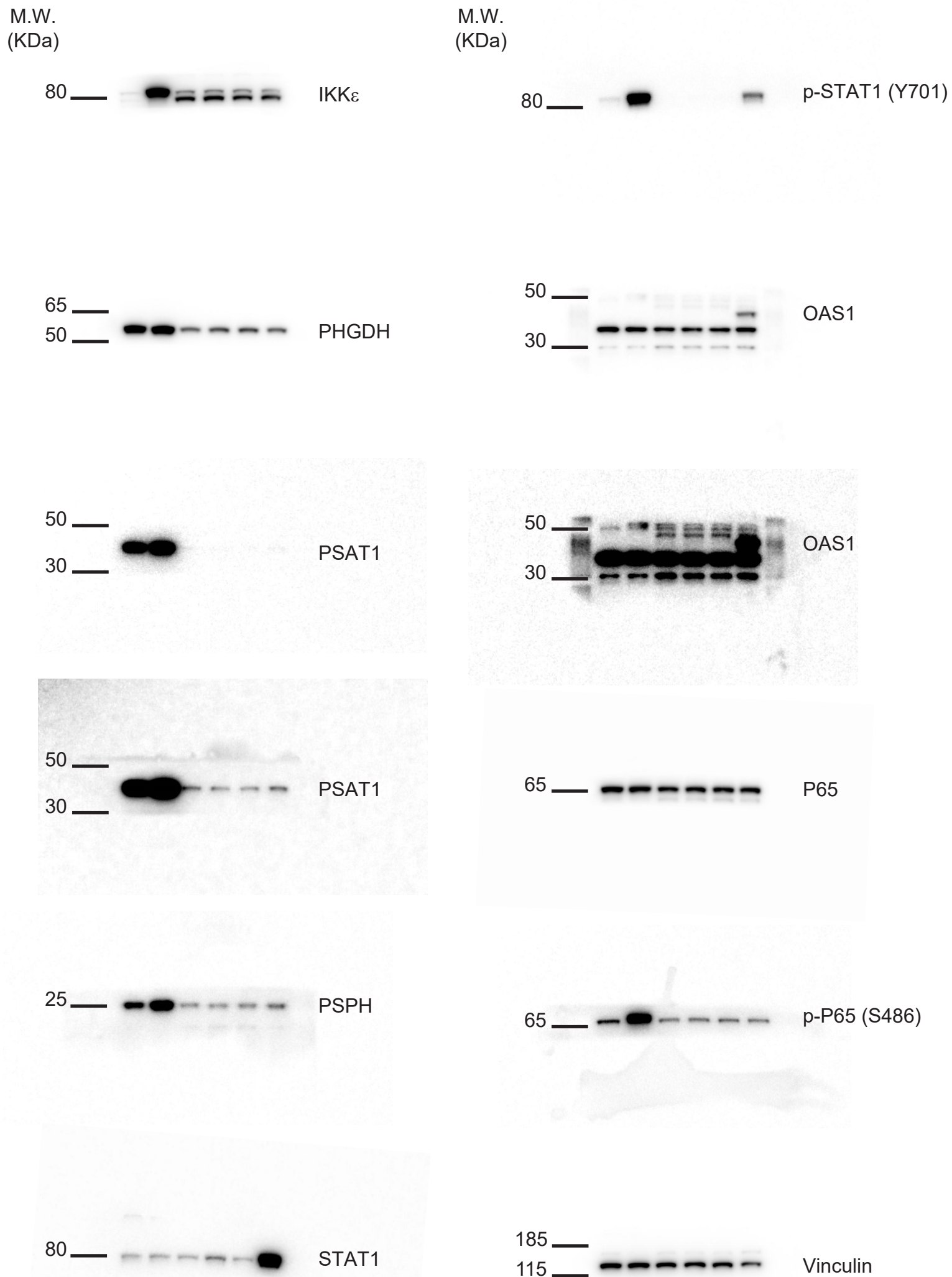

Figure EV5C

Supplement: Supplementary file 4 — Source Data for Expanded View [file EMBR-21-e48260-s010.zip › EV_Figure_Source_Data/FigureEV4/Figure EV4 Panel C.pdf]

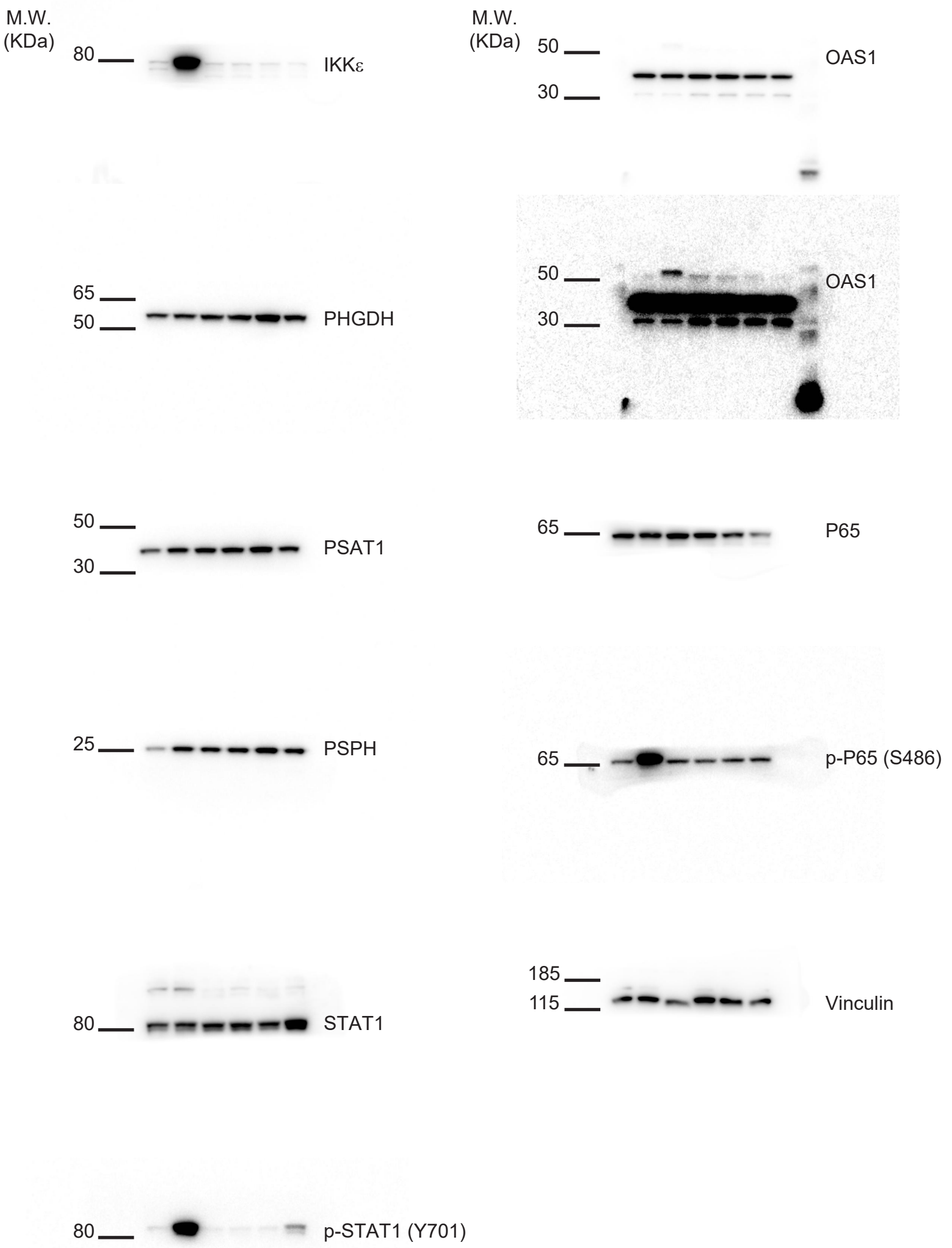

Figure EV5A

Supplement: Supplementary file 4 — Source Data for Expanded View [file EMBR-21-e48260-s010.zip › EV_Figure_Source_Data/FigureEV4/Figure EV4 Panel A.pdf]

M.W.  
(KDa)

80 —

65 —

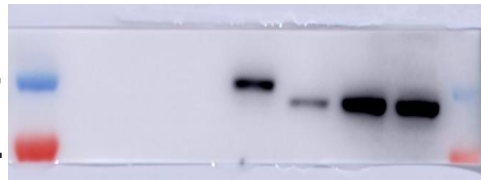

IKKε

65 —

50 —

30 —

25 —

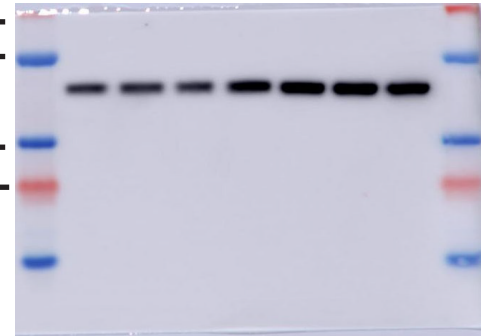

Actin

Supplement: Supplementary file 6 — Source Data for Figure 1 [file EMBR-21-e48260-s004.zip › Figure 1/Figure 1 Panel A.pdf]

M.W.  
(KDa)

115 —  
80 —  
65 —

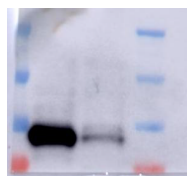

IKKε

65 —  
50 —  
30 —  
25 —

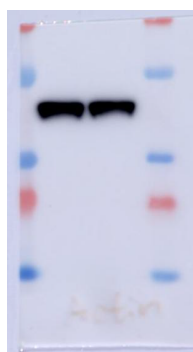

Actin

Supplement: Supplementary file 7 — Source Data for Figure 2 [file EMBR-21-e48260-s005.zip › Figure 2/Figure 2 Panel B.pdf]

M.W.  
(KDa)

185 —

115 —

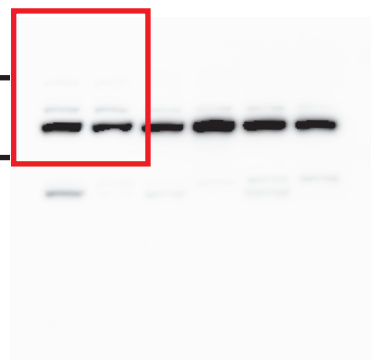

Vinculin

M.W.  
(KDa)

65 —

50 —

30 —

25 —

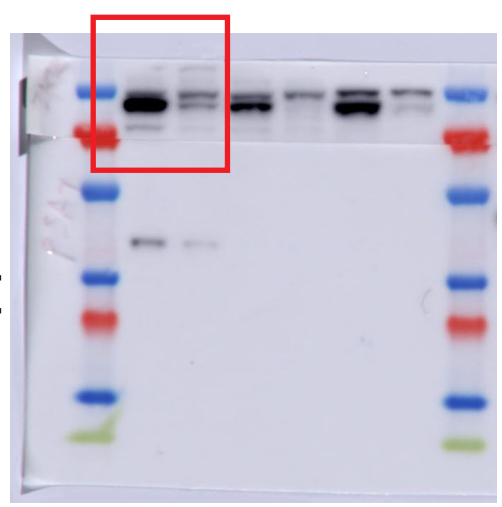

IKKε

Supplement: Supplementary file 7 — Source Data for Figure 2 [file EMBR-21-e48260-s005.zip › Figure 2/Figure 2 Panel A.pdf]

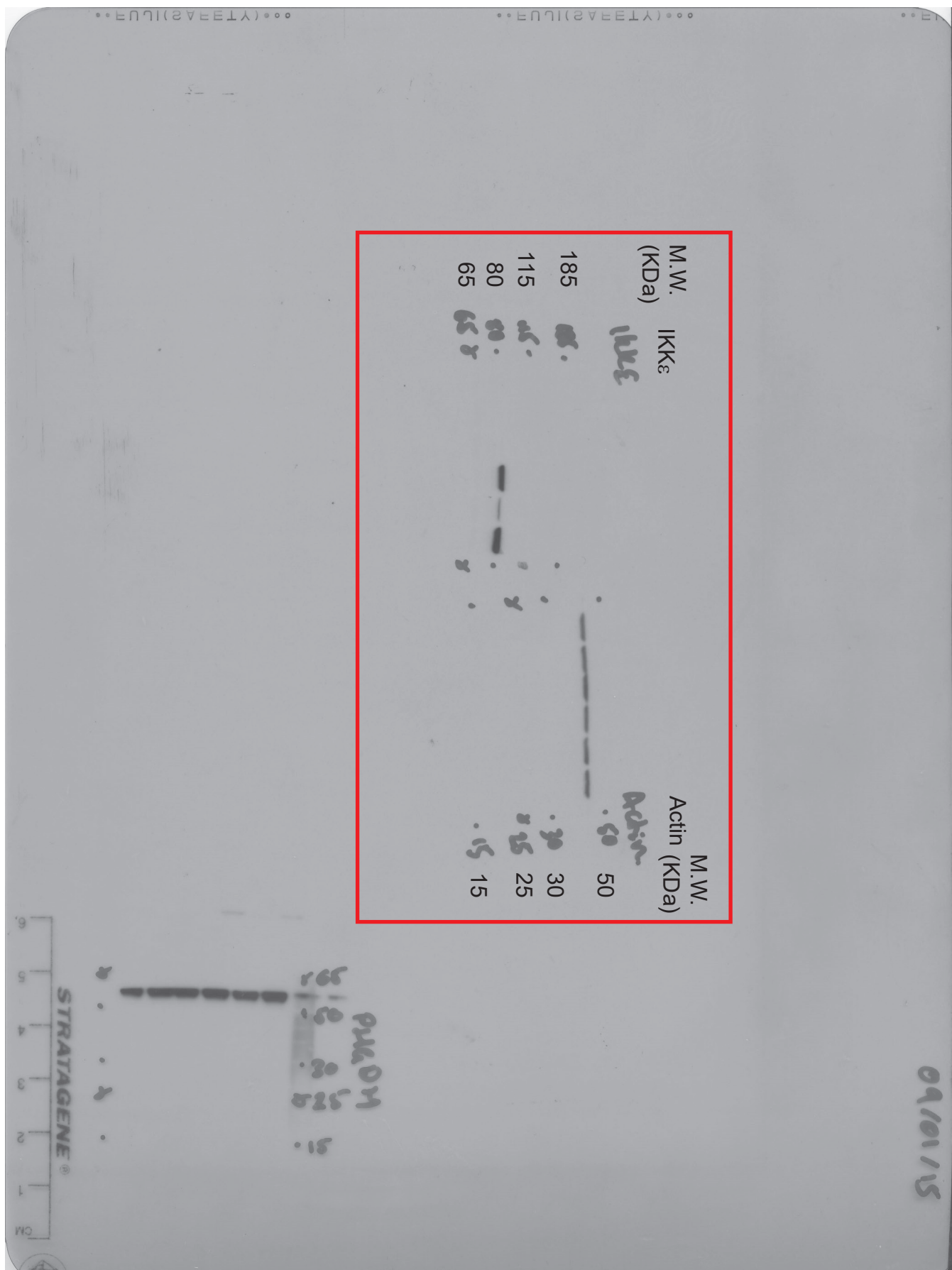

Figure 3F

Supplement: Supplementary file 8 — Source Data for Figure 3 [file EMBR-21-e48260-s006.zip › Figure 3/Figure 3 Panel F.pdf]

M.W.  
(KDa)

115  
80

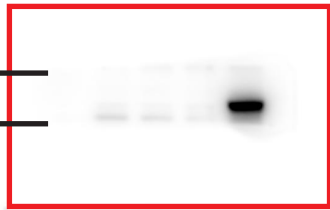

IKK $\epsilon$

M.W.  
(KDa)

25

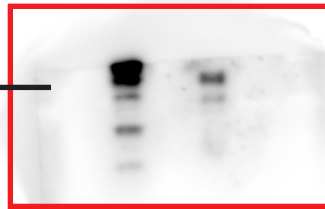

HA-tag (GFP)

65  
50

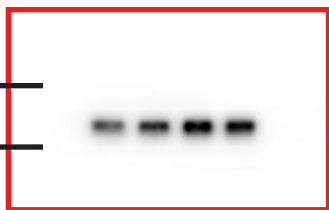

PHGDH

50  
30

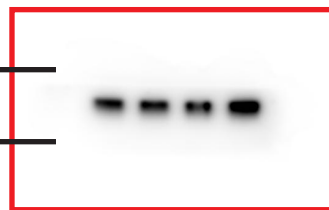

PSAT1

25

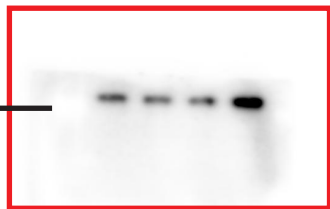

PSPH

50

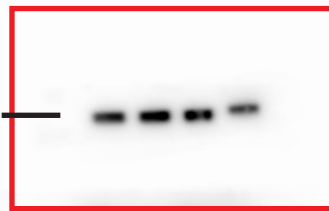

IRF3

p-IRF3 (S396)

50

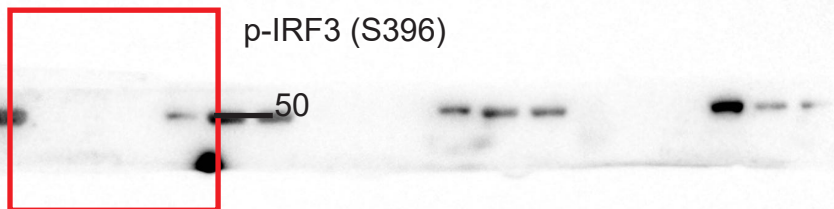

185  
115

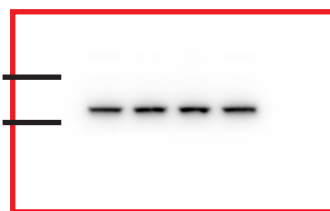

Vinculin

Figure 4C

Supplement: Supplementary file 9 — Source Data for Figure 4 [file EMBR-21-e48260-s007.zip › Figure 4/Figure 4 Panel C.pdf]

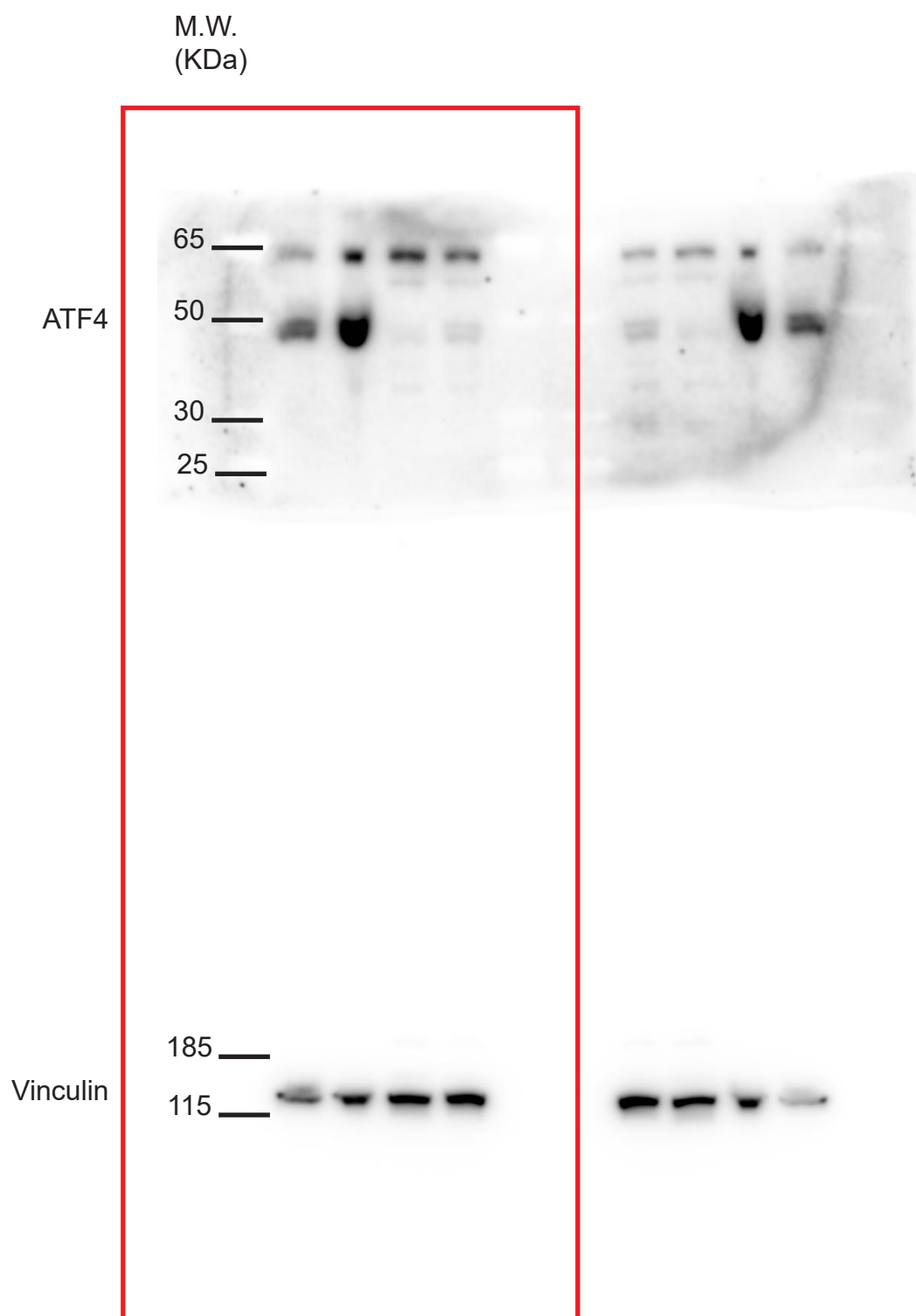

Figure 4A

Supplement: Supplementary file 9 — Source Data for Figure 4 [file EMBR-21-e48260-s007.zip › Figure 4/Figure 4 Panel A.pdf]

M.W.  
(KDa)

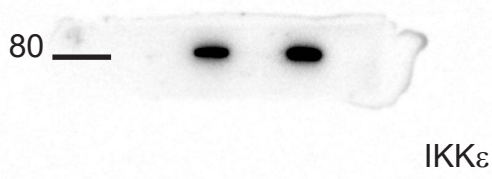

M.W.  
(KDa)

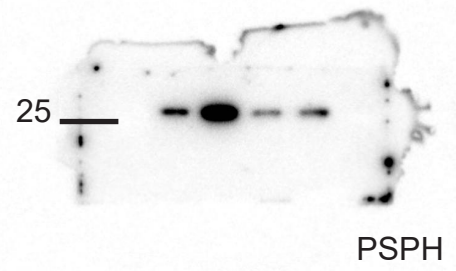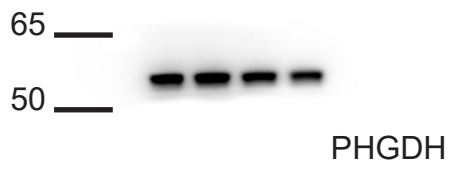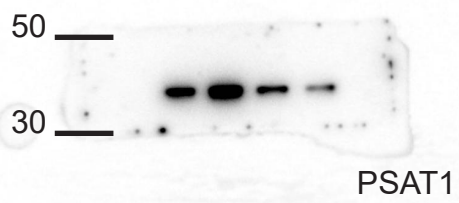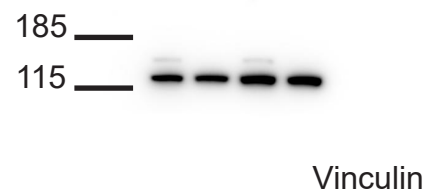

Figure 4E

Supplement: Supplementary file 9 — Source Data for Figure 4 [file EMBR-21-e48260-s007.zip › Figure 4/Figure 4 Panel E.pdf]

M.W.  
(KDa)

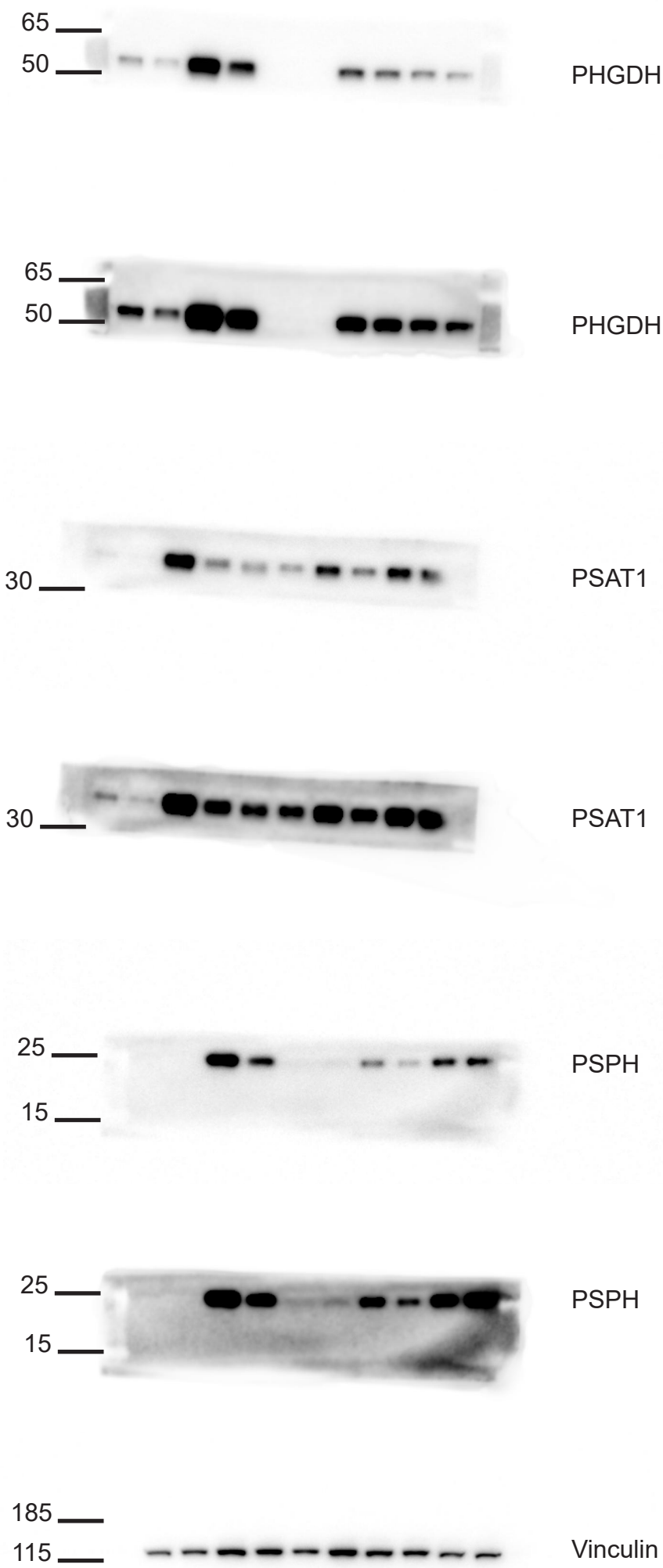

Figure 5G

Supplement: Supplementary file 10 — Source Data for Figure 5 [file EMBR-21-e48260-s008.zip › Figure 5/Figure 5 Panel G.pdf]

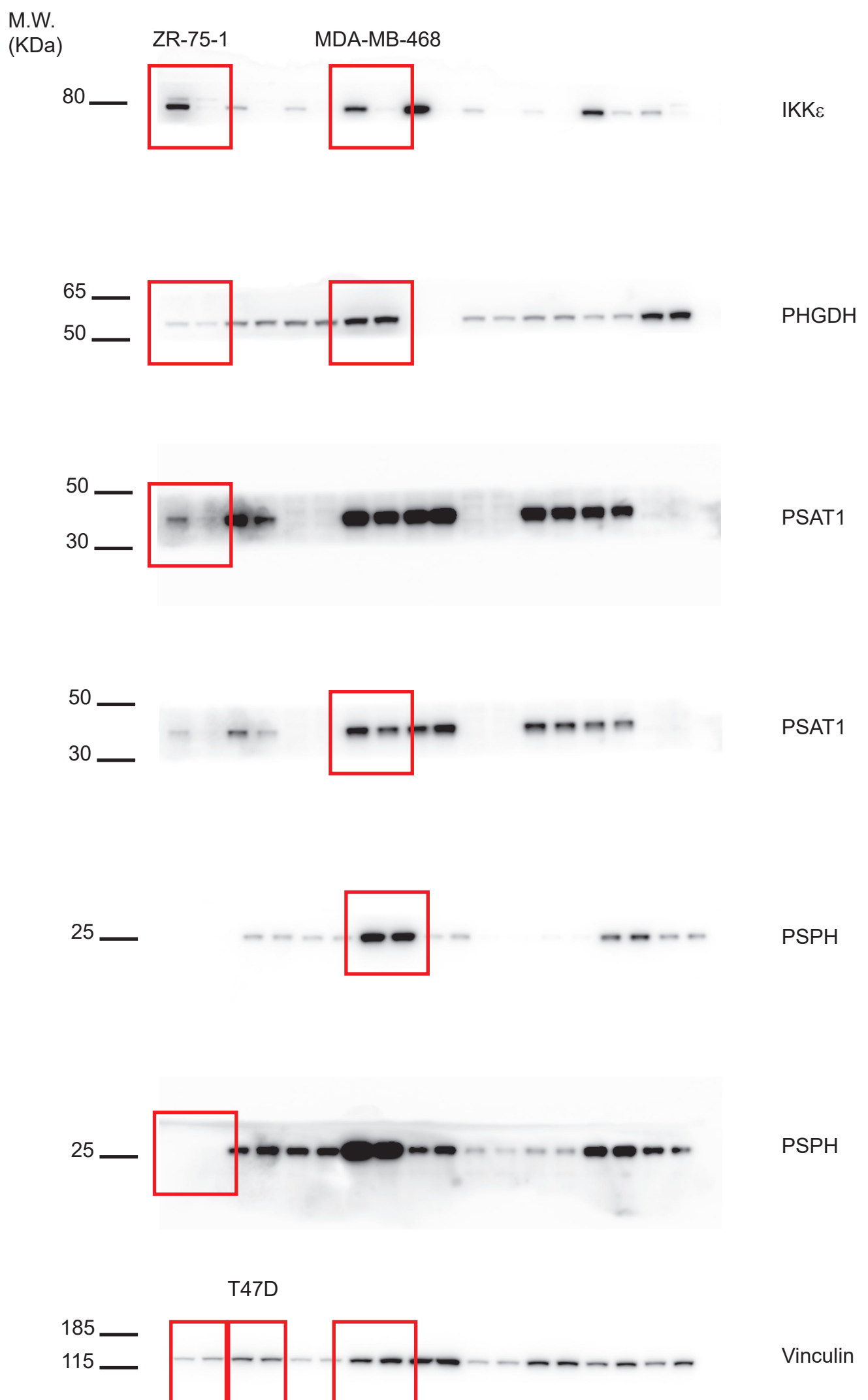

Figure 5B

Supplement: Supplementary file 10 — Source Data for Figure 5 [file EMBR-21-e48260-s008.zip › Figure 5/Figure 5 Panel Bi.pdf]

M.W.  
(KDa)

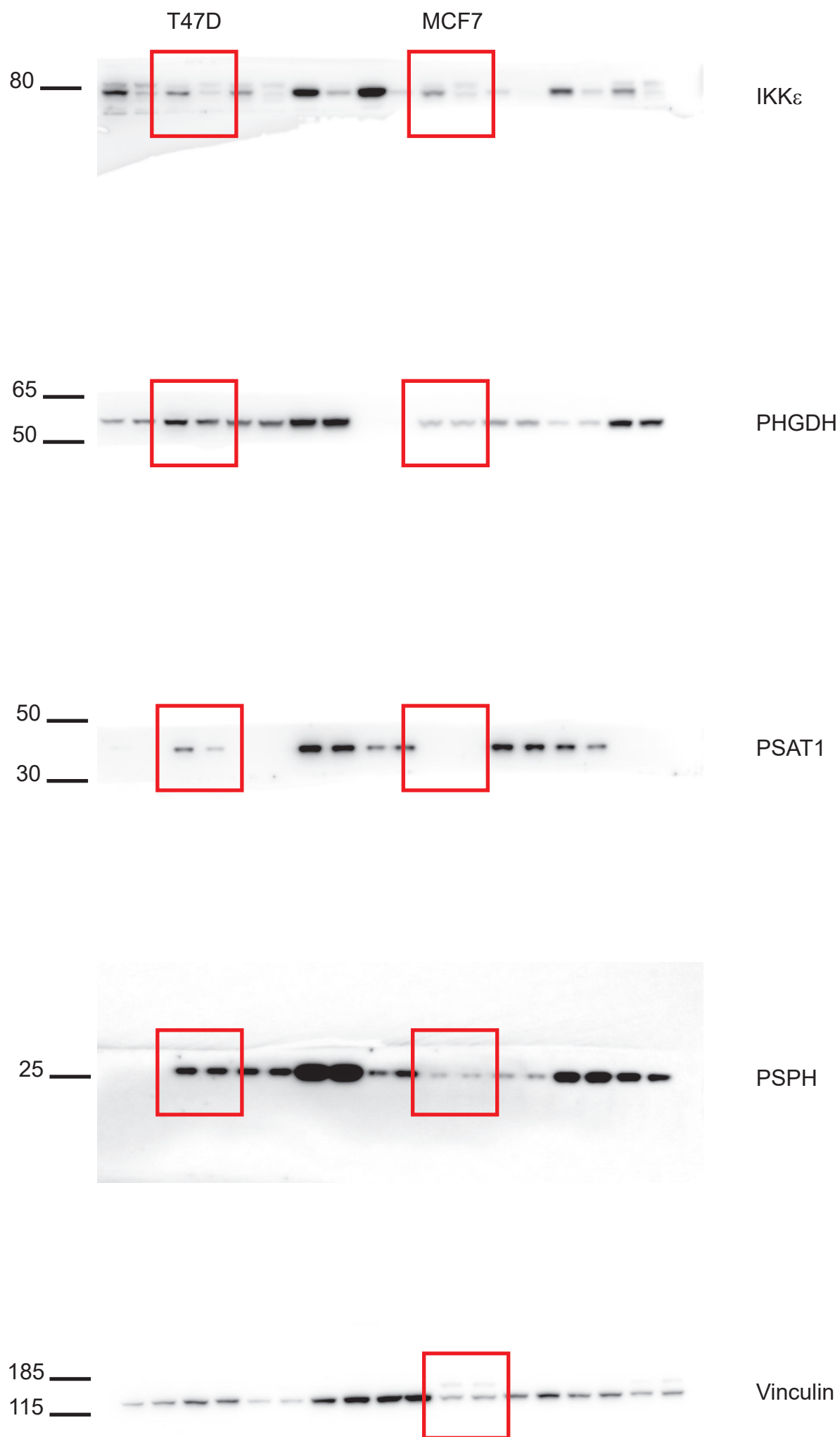

Figure 5B

Supplement: Supplementary file 10 — Source Data for Figure 5 [file EMBR-21-e48260-s008.zip › Figure 5/Figure 5 Panel Bii.pdf]
